# Caring in crisis: family dynamics and child wellbeing in Rohingya refugee camps

**DOI:** 10.3389/fpsyg.2025.1685206

**Published:** 2026-03-02

**Authors:** Bree Akesson, Ashley Stewart-Tufescu, Cindy Sousa, Karen Frensch

**Affiliations:** 1 Faculty of Social Work, Wilfrid Laurier University, Brantford, ON, Canada; 2 Faculty of Social Work, University of Manitoba, Winnipeg, MB, Canada; 3 School of Social Work & Criminal Justice, University of Washington, Tacoma, WA, United States

**Keywords:** Rohingya refugee, perinatal wellbeing, family resilience, parenting, humanitarian crisis, displacement

## Abstract

For Rohingya families living in the world’s largest refugee camps, the perinatal period unfolds under conditions of profound insecurity and deprivation, despite being a critical developmental window that reshapes family dynamics and lays the foundation for child outcomes. Guided by the World Health Organization (WHO) Nurturing Care Framework—which emphasizes the interdependence of health, nutrition, responsive caregiving, early learning, and safety—we conducted collaborative family interviews, semi-structured interviews with mothers and fathers, and Life Story Board activities with school-aged children across 32 families to examine how caregiving is negotiated during this sensitive period. Findings revealed three interconnected themes: (1) parent–child wellbeing strained by chronic stress, trauma, and structural constraints; (2) intensified resource scarcity that heightens worries about food, healthcare, education, and equitable caregiving; and (3) shifting family dynamics in which older siblings—especially girls—assume significant caregiving and economic roles as parental capacities are stretched. Despite these pressures and clear gaps across the nurturing care domains, families demonstrated notable adaptability, moral commitment, and resilience in supporting their children. The results underscore the urgent need for holistic, contextually grounded interventions that bolster caregiver wellbeing, address structural barriers, and strengthen family systems in protracted displacement settings.

## 1 Introduction and literature review

### 1.1 Parenting and early childhood development

Parental wellbeing is foundational for responsive caregiving, particularly in contexts marked by adversity, displacement, and chronic resource insecurity. Parents’ capacity to care for their children is not only shaped by individual psychological resilience but also by the broader socio-political environment in which caregiving occurs. In situations of political violence, disruptions to parents’ mental health—such as chronic stress, fear, and trauma—can impair their ability to respond sensitively and consistently to their children’s needs. Parental distress contributes to recursive cycles of diminished caregiving, where children’s behavioral difficulties further exacerbate parents’ own sense of helplessness and fatigue. Moreover, economic precarity adds another layer of psychological strain, as refugee parents may struggle to provide basic necessities, leading to guilt, emotional withdrawal, and increased risk of intergenerational trauma (Akesson and Badawi, 2021). Together, research suggests that interventions focused on child development must simultaneously attend to the mental health and material wellbeing of caregivers with a particularly keen eye to the complications of caregiving within contexts of war and displacement, as these complications are inextricably linked to the quality of care that parents can provide.

### 1.2 The Rohingya crisis and challenges to family life

The Rohingya, a predominantly Muslim ethnic minority group from Myanmar’s Rakhine State, have endured decades of systemic persecution and statelessness. Denied citizenship under Myanmar’s 1982 Citizenship Law, the Rohingya have been excluded from legal recognition and stripped of basic rights such as freedom of movement, access to education, and participation in public life (Southwick, 2015). This legal exclusion has been reinforced by targeted campaigns of violence against the Rohingya, culminating in the genocidal military assaults of 2017, during which entire Rohingya villages were burned, civilians were raped and killed, and over 700,000 Rohingya were forcibly displaced into neighboring Bangladesh (Fortify Rights, 2018; United Nations High Commissioner for Refugees (UNHCR), 2023). These events have been widely recognized as crimes against humanity and acts of domicide or the deliberate destruction of home as a strategy of erasure and ethnic cleansing (Akesson and Basso, 2022).

Since their mass displacement in 2017, most Rohingya have remained confined within the refugee camps of Cox’s Bazar, Bangladesh, which is now home to the largest stateless population in the world. Despite Bangladesh’s willingness to host Rohingya refugees, families live under conditions of protracted encampment marked by spatial restriction, limited autonomy, and legal invisibility. Refugees are prohibited from formal employment, accessing national education systems, or integrating with host communities (Milton et al., 2017). These limitations reinforce a state of perpetual displacement and undermine family and community cohesion.

The challenges to family life within the camps are manifold. One of the most persistent threats is food insecurity. Despite humanitarian aid, the World Food Programme has been forced to reduce monthly food rations to $8 per person due to funding shortfalls (Mones et al., 2023). This has significantly impacted child health and nutrition, with recent surveys indicating chronic malnutrition and stunting well above emergency thresholds (IPC, 2025). Families face daily struggles to obtain adequate nutrition, often relying on nutrient-poor diets with limited cooking fuel, which in turn places added stress on caregivers.

Education is another domain where Rohingya families face systemic exclusion. For years, formal education beyond Level 3 was prohibited by Bangladeshi authorities, a policy grounded in the belief that repatriation should be the long-term solution (Hossain, 2023). Although the Myanmar curriculum has been introduced in limited pilot projects in the camps, implementation remains sparse, with significant gender disparities in access. Girls in particular are often withdrawn from learning spaces due to caregiving duties, child marriage, or concerns about safety and dignity (Plan International, 2018). The absence of educational pathways deprives children of the tools necessary for future rebuilding.

Healthcare and protection services are similarly fragile. Rohingya families rely heavily on overburdened humanitarian systems for primary healthcare, with limited access to specialized services such as maternal and newborn care, mental health, or disability support (World Health Organization (WHO), 2023). Pregnant women often face heightened vulnerabilities due to overcrowded living conditions, loss of traditional birthing support systems, and physical insecurity. Intra-camp violence—including extortion, gender-based violence, and the rise of armed groups—undermines protection systems and imposes additional risks, especially for women and girls (ACAPS, 2023; UN Women, 2025).

These daily hardships are rooted in the broader denial of rights and resources that defines the Rohingya experience (Uddin, 2020). As Southwick (2015) and the United Nations (UN) (2018) have argued, the Rohingya crisis is not only a humanitarian catastrophe but also a politically orchestrated campaign of exclusion, erasure, and containment. Even in displacement, Rohingya families are subject to securitized and restrictive aid regimes, where basic services are conditional, and repatriation is continuously discussed despite conditions in Myanmar being increasingly untenable. Such systematic and structural violence erodes not only material wellbeing but also the dignity and coherence of family life.

Rohingya families navigate a layered landscape of dispossession: from the trauma of generational statelessness, surviving genocide, and the subsequent forced displacement to the ongoing restrictions of life in Bangladesh’s refugee camps. Parenting and caregiving under such conditions require immense perseverance, which is consistently undermined by legal invisibility, inadequate services, discriminatory camp policies, and sociopolitical marginalization.

### 1.3 Parenting in contexts of political violence

Parenting under conditions of political violence presents unique and often overwhelming challenges that reverberate across generations. Political violence disrupts the social, cultural, economic, and psychological environments in which caregiving takes place, resulting in diminished parental efficacy, increased mental health burdens, and dangerous caregiving practices. Rather than being a temporary disruption, such violence produces a circuitous and compounding pattern of stress and caregiving breakdown, whereby parental distress leads to diminished caregiving, which in turn exacerbates child distress, reinforcing the cycle of family suffering.

Empirical research underscores the destabilizing effects of war and political violence on parenting capacity. Political violence has been associated with elevated levels of parental depression, anxiety, post-traumatic stress disorder (PTSD), and feelings of helplessness—all of which compromise parents’ ability to respond sensitively to their children’s needs (Betancourt, 2015; Betancourt et al., 2015; Panter-Brick et al., 2014). As Sousa et al. (2024) discuss, these emotional and psychological disruptions are not only internalized by parents but are often transferred to their children through harsh, inconsistent, or withdrawn caregiving. In the context of prolonged societal stress, Chasson et al. (2025) found that mothers of young children frequently experienced intrusive thoughts and dissociative symptoms that disrupted their ability to bond and respond effectively to their infants. Similarly, research on war-exposed populations has shown that maternal trauma and dissociation are linked with more disorganized caregiving representations and lower-quality mother–infant interactions (Isosävi et al., 2020).

This erosion of parental functioning under political violence unfolds in circular and self-reinforcing ways. As highlighted by El-Khani et al. (2016), the stressors associated with displacement—such as poverty, insecurity, social isolation, and legal precarity—can degrade parenting capacity over time. Parents may become more irritable, emotionally withdrawn, or reliant on punitive forms of discipline. These behavioral shifts negatively affect children’s emotional security and wellbeing, leading to increased child distress, behavioral issues, and trauma symptoms (Maddah et al., 2024; Masten and Narayan, 2012). For example, Lahti et al. (2019) found that Palestinian mothers exposed to war trauma reported heightened emotional reactivity to infant crying, which in turn was linked to negative maternal self-perceptions and greater parental helplessness. In turn, children’s difficulties can amplify parental feelings of inadequacy and despair, thus perpetuating a cycle in which both parent and child suffer without adequate intervention (Cummings et al., 2009). Importantly, even in these extreme conditions, parents do not uniformly collapse under stress. Many caregivers actively adapt by developing protective, compensatory and resilient practices, including increased family communication, moral guidance, spiritual engagement, and future-oriented thinking (Akesson et al., 2026; Betancourt and Khan, 2008; Walsh, 2016). These efforts reflect amplified caregiving, where caregiving becomes an intentional act of resistance and survival amid adversity. For instance, Akesson and Sousa (2020) found that refugee parents in Lebanon often sought to comfort and distract their children during times of fear, using storytelling or humor as coping mechanisms representing small yet meaningful acts that contributed to family resilience and parental empowerment.

Nevertheless, such individual efforts can only go so far without broader systemic and structural support. As Gavidia-Payne et al. (2015) argue, parental resilience is not merely an internal trait but a context-dependent process, shaped by access to resources, legal protections, and supportive community environments. In contexts of protracted displacement or state violence, where such conditions are systematically denied, even the most committed parents face limits in their ability to maintain responsive caregiving.

Ultimately, parenting in contexts of political violence cannot be separated from the larger structural conditions in which it occurs. Political violence initiates a recursive cycle of trauma, stress, and caregiving breakdown that deeply affects both caregivers and children. Without sustained, multilevel interventions that address both caregiver wellbeing and the systemic and structural barriers, these cycles are likely to persist across generations. Recognizing and interrupting this cycle is a critical step toward supporting family resilience and child development in contexts of war and displacement.

### 1.4 Parental resilience in adversity

While parenting in contexts of political violence and displacement is marked by profound challenges, research increasingly points to the extraordinary resilience demonstrated by caregivers under siege. Far from passive victims, many parents adapt their caregiving strategies with amplified warmth, heightened vigilance, compensatory strategies, and intentional efforts to sustain emotional connection and protect their children. This kind of “resilience through caregiving” highlights how parenting itself can become a vehicle for survival, meaning-making, and hope in times of crisis (Stevenson, 2011).

The concept of parental resilience has evolved beyond static trait-based definitions to encompass a dynamic, relational process shaped by risk exposure, available supports, and individual and cultural meaning systems. Gavidia-Payne et al. (2015) define parental resilience as “a parent’s ability to manage and adapt to adversity while maintaining their caregiving responsibilities” (p. 112). This definition foregrounds the balance parents must strike between their own distress and meeting their children’s needs—a tension intensified in contexts of armed conflict or forced migration. Sousa et al. (2024) extend this framing by emphasizing the embedded nature of resilience: rather than an internal trait, it emerges from caregivers’ capacity to draw on personal, social, and structural resources to sustain caregiving in adverse settings.

In war-affected contexts, this resilience is often expressed through amplified caregiving behaviors, such as increased affection, emotional attunement, and physical closeness. Studies among Syrian and Palestinian refugee populations have documented how parents double down on expressions of love and protection in response to violence and displacement, often using storytelling, play, or spiritual practices to soothe and connect with their children (El-Khani et al., 2016; Panter-Brick et al., 2014). These behaviors are not merely reactive but strategic and symbolic; they assert a moral order in which children are still worthy of care, structure, and joy even when the external world denies them safety or dignity. In the Rohingya context, families demonstrate “resilience through caregiving,” adapting roles and responsibilities, drawing on spiritual practices, and improvising forms of protection in the absence of formal support. These adaptive strategies such as storytelling, communal prayer, and improvised routines align with the principles of responsive caregiving, yet they emerge under radically different conditions in contexts of political violence.

Such intentional caregiving also plays a protective role in buffering children from trauma. Masten and Narayan (2012) describe caregiving as a “protective system” that mitigates the effects of stress and uncertainty by offering children consistent routines, emotional regulation, and a sense of security. For example, research by Thabet et al. (2009) in Gaza found that children whose parents maintained positive coping strategies and family routines exhibited better emotional and behavioral outcomes, even amid chronic exposure to political violence. This suggests that parental resilience operates not only as self-preservation but as intergenerational buffering, allowing families to maintain coherence and function despite disruption. Within this context, caregiving itself becomes both an act of survival as well as a site of exhaustion. Sousa et al. (2024) note that in politically violent settings, caregivers often operate in compressed caregiving ecologies—spaces where resources are minimal, social supports are fragmented, and institutional trust is low.

Importantly, resilience through caregiving is not universally available or equally supported. The ability of parents to maintain caregiving roles under pressure is deeply shaped by structural conditions, including access to shelter, food, healthcare, psychosocial support, and legal protection (Tol et al., 2013). In the absence of such supports, even the most devoted parents may find their caregiving capacity diminished. This highlights the critical role of humanitarian and policy interventions in not just targeting children, but supporting caregivers as frontline agents of resilience (Leckman et al., 2014).

Gender norms also shape how resilience is expressed across families in crisis settings. Although research has traditionally focused on mothers, a growing evidence base highlights the vital yet often overlooked contributions of fathers and extended family members in humanitarian contexts. Father involvement in perinatal care is consistently associated with improved maternal health, reduced preterm labor, better birth outcomes, and lower rates of postpartum depression, with fathers’ emotional and practical support strengthening maternal empowerment and overall family wellbeing (Firouzan et al., 2018; Lee et al., 2018; Small et al., 2025; Xie et al., 2023). At the same time, fathers themselves face heightened risk of perinatal mental health difficulties (Darwin et al., 2021; Watkins et al., 2024; Xie et al., 2023). These risks are amplified in humanitarian contexts where displacement, cultural dislocation, insecure livelihoods, and limited institutional support can undermine their caregiving roles (Gopal et al., 2020; Mestre et al., 2022; Musiwa et al., 2024). Perinatal services frequently exclude fathers, treating them as secondary caregivers, constrained further by traditional gender norms and inflexible provider attitudes (Buek et al., 2021; Gopal et al., 2020; Musiwa et al., 2024; Small et al., 2025; Walsh et al., 2021). Yet the limited evidence from humanitarian settings shows that fathers often adapt in creative ways (Akesson et al., 2020; Gokani et al., 2015, 2016). Recognizing these dynamics broadens narrow conceptions of resilience and foregrounds the diverse ways families mobilize care under conditions of displacement.

Ultimately, the study of parental resilience in adversity reveals not only the burdens parents carry, but also the profound moral commitments they enact. As Sousa et al. (2024) argue, caregiving under siege becomes an ethical act: a refusal to abandon one’s role as protector, nurturer, and guide, even when systems of support have collapsed. Recognizing and strengthening this resilience requires approaches that are relational, ecological, and justice-oriented, affirming the dignity and agency of parents who continue to care for their children despite impossible odds.

## 2 The WHO Nurturing Care Framework in contexts of war and displacement

The World Health Organization’s Nurturing Care Framework (NCF) (World Health Organization (WHO), 2018) understands early childhood development through five interrelated components: good health, adequate nutrition, responsive caregiving, opportunities for early learning, and safety and security. At its core, the NCF positions parents and caregivers as central to children’s developmental wellbeing, recognizing that responsive caregiving—marked by attentiveness, sensitivity, and emotional attunement—is foundational to healthy growth.

Crucially, the NCF highlights the period from pregnancy to age three as the most critical window for brain development, with 80% of the brain formed by age three and early experiences shaping lifelong health, learning, and wellbeing. This is particularly salient during the perinatal period, when maternal physical and mental health, nutrition, and access to care directly affect both immediate neonatal outcomes and long-term child development trajectories (World Health Organization (WHO), 2018). In this sensitive window, interventions such as skin-to-skin contact, breastfeeding support, maternal mental health services, and protection from violence and environmental hazards are not optional but foundational to the enabling nurturing environments envisioned by the NCF.

In our work, we integrate the holistic family NCF model with our theoretical framework that honors the conditions within which nurturing care unfolds. In humanitarian and crisis-affected contexts, nurturing care is not merely difficult to provide; it is politicized, conditional, and deeply constrained by political and structural violence, including that which defines protracted displacement. Violent and repressive environments drastically undermine parenting, creating crises in parents’ caregiving and supervisory practices, as well as their sense of parental efficacy and their social and psychological wellbeing (Sousa et al., 2024). As such, our critical theoretical lens takes into account not only the importance of all the parental tasks of nurturing care (World Health Organization (WHO), 2018), but also the social and political environments that surround these practices for refugees.

Indeed, among the Rohingya in Bangladesh’s refugee camps, conditions for nurturing care are profoundly shaped by genocide, forced migration, and ongoing restrictions on movement, education, healthcare, and livelihoods. Environmental hazards such as flooding, landslides, and fires exacerbate material deprivation, while overcrowding, food insecurity, and intra-camp violence compromise safety and increase caregiver stress. For pregnant women and new mothers, these insecurities are compounded by limited access to skilled birth attendants, antenatal and postnatal care, and psychosocial support, creating profound risks for both maternal and infant health.

Within such compressed caregiving ecologies where resources are minimal, social supports fragmented, and institutional trust low, the ability to provide consistent, responsive care depends on both personal resilience and the availability of structural supports (Murphy et al., 2017; Sousa et al., 2024). Mothers and other caregivers are expected to remain emotionally available while simultaneously managing trauma, hunger, and anticipatory grief about their children’s future, conditions that stand in sharp contrast to the kinds of idealic environments required for and envisioned by the NCF.

Like other conceptions of the importance and role of caregiving for determining child health and development, the NCF is a valuable tool. But, as we describe here, it must be applied with a commitment to contextual nuance that understands caregiving as inseparable from the conditions that surround it. NCF’s five components can guide humanitarian responses, provided they are adapted to reflect the complex realities of war-affected families and the distinct needs of the perinatal period in contexts of war, displacement, and genocide. Examples include initiatives (both research and practice) that promote health by integrating maternal and newborn care with mental health and psychosocial support, particularly for caregivers; supporting adequate nutrition through food security interventions tailored to resource-poor settings and sensitive breastfeeding promotion that honors the ways this practice is often disrupted during violence and displacement; promoting responsive caregiving through culturally relevant parent-support programs that address trauma and build confidence in newborn care among caregivers whose parental role has been profoundly interrupted and undermined by violence and oppression; creating opportunities for early learning through family-based stimulation and play that starts in pregnancy, involves all caregivers, and acknowledges the difficultly of educating and playing when one is dealing with complex and ongoing trauma and loss (including violent loss of the resources parents may traditionally utilize were it not for the political upheavel); safeguarding caregiver wellbeing by mitigating violence, ensuring privacy for breastfeeding, and reducing environmental hazards in shelters.

In the Rohingya context, adapting the NCF also requires addressing systemic barriers—such as restrictive camp policies that do not allow families to leave the camps, earn a living, or engage in formal schooling beyond Grade 3—and leveraging existing family and community strengths, including those that may have been amplified through the tragedies they have faced, like intergenerational caregiving, faith practices, and storytelling. Implementing the NCF meaningfully during the perinatal period therefore demands interventions that are technically sound, trauma informed, and politically aware, advocating for refugee rights while supporting caregivers as frontline agents of survival and development. In other words, parents are people who also need nurturing care at the same time as their children.

In sum, while the World Health Organization (WHO) (2018) Nurturing Care Framework provides a vital blueprint for early childhood development, its application in crisis contexts must expand to recognize caregiving in the perinatal period as both a personal and political act, embedded in histories of war, displacement, and structural exclusion. This shift—from apolitical and ahistorical deficits-based models to politically and historically situated approaches that affirm caregiver dignity, agency, and expertise—can better align global frameworks with the everyday realities and demonstrated resilience of families caring under siege.

The literature underscores that the perinatal period is a uniquely sensitive time for both children’s development and family wellbeing. For Rohingya families in Bangladesh’s refugee camps, this period unfolds under conditions of extreme adversity, all of which directly constrain the five components of nurturing care. While global frameworks such as the NCF provide essential guidance, there remains a critical need for research that identifies and develops evidence-informed, culturally relevant interventions tailored to the realities of protracted displacement. Addressing these gaps requires approaches that listen closely to the realities and experiences of caregivers, and not only support early child development but also strengthen the capacity of traumatized caregivers and family systems to adapt and thrive. This study responds to that need by exploring the everyday experiences of Rohingya families during the perinatal period, generating knowledge that can inform collaborative, contextually grounded solutions. Building such solutions depends on meaningful, sustained partnerships between researchers and refugee communities. These partnerships should be grounded in mutual trust, cultural understanding, and shared goals for the wellbeing of mothers, infants, families, and societies who have been targeted and oppressed.

## 3 Materials and methods

### 3.1 Developing community-researcher partnerships

Strong community partnerships are essential for conducting international research in volatile settings. With its focus on women and families during the perinatal period and strong track record as a respected community organization, Hope Foundation for Women and Children of Bangladesh serves as the primary community partner for this project. Situated in Cox’s Bazar district, Hope Foundation serves between 800 and 1,500 patients per day (Cleland, 2017). In 2020 alone, Hope Foundation assisted 12,360 women through the Safe Motherhood program and participated in 3,844 births (Hope Foundation, 2021).

At the outset of the project, the first and second authors traveled to Bangladesh for a two-week field visit to solidify the research partnership with Hope Foundation. During this field visit, we met with Hope Foundation research staff and key community stakeholders to discuss participant recruitment, compensation for participants, contextually relevant methodology, and effective dissemination of findings to ensure maximum impact on the population of interest. We worked closely with three Rohingya cultural liaisons with specialized knowledge of the Rohingya culture and norms. We piloted our data collection methods to ensure cultural relevance and contextual feasibility. The methodology was subsequently refined based on the pilot data gathering experience.

Hope Foundation played a pivotal role in recruiting and engaging with families during the perinatal period. A community health worker from Hope Foundation’s Field Hospital informed Rohingya families expecting the birth of a child (approximately six-months gestation) about the study and asked families if they would agree to meet with researchers to hear more about how to participate. The first and second authors then met with families who expressed an interest in the study to explain the research procedures and obtain informed consent from all family members. All 32 families that researchers spoke to agreed to participate in the study.

Prior to data collection, all research team members, including student research assistants, transcriptionists, and cultural liaisons, were trained on the purpose of the study, interview protocols, and ethical considerations. This training was provided by the first and second authors, who bring extensive experience working with cross-language translation and transcription teams in research with war-affected and displaced populations (Akesson et al., 2018; Akesson and Coupland, 2020).

### 3.2 Gathering research data

The research used a longitudinal interpretive phenomenological analysis (LIPA) design. LIPA emphasizes the changing meaning of an experience (Smith et al., 2009) and is therefore well-positioned to explore temporal events (McCoy, 2017) such as pregnancy, childbirth, and postpartum childrearing. To gather our research data, we interviewed each family when the mother was approximately six months gestation (Time 1, October 2024). (Note: Longitudinal data collection continued in 2025 with two additional data collection time points: Time 2 (April 2025) at three to six months postpartum and Time 3 (October 2025) at nine months to one year postpartum, a time that marks the baby’s transition from infancy to toddlerhood. However, for this article, we only present findings from Time 1 data when families were anticipating the birth of their child.)

Data collection methods included collaborative family interviews (CFI), individual semi-structured interviews (SSIs) with mothers and fathers, and play-based interviews with school-aged children in the family using the Life Story Board (LSB). Each method is described in greater detail below.

#### 3.2.1 Collaborative family interviews (CFIs)

We asked families to participate in a 45 minute collaborative family interview (CFI), each considering the unique trajectory of the family and how they experience parenting within their social, cultural, and political situations. CFIs engage multiple family members in the research process providing the research team with the opportunity to observe family dynamics such as mother–father communication and parenting strategies. The first author developed, piloted, and revised this method over the past decade, and it has been used successfully with war-affected families in a range of discplacement contexts (Akesson, 2011, 2014). Interviews were conducted by either the first or second author, who were accompanied by at least one of three Rohingya cultural liaisons. The CFI focused on cultural practices during pregnancy, social supports, and the psychosocial wellbeing of the expectant mother, father, and other family members, and hopes and concerns for the new baby. We also gathered basic family demographics, including circumstances of flight from Myanmar, length of time in Bangladesh, family composition and characteristics, living situation, economic status, and details about pregnancy (including number of pregnancies, number of pregnancy losses if any, access to prenatal care, etc.). Questions were designed to encourage reflection while providing space for families to feel control over what aspects they chose to discuss, which is crucial when conducting research on sensitive topics (Akesson et al., 2018; Meredith et al., 2017).

#### 3.2.2 Semi-structured interviews (SSIs) with mothers and fathers

We conducted separate semi-structured interviews (SSIs) lasting approximately 60 minutes with mothers and fathers individually. SSIs provided a more dedicated opportunity to explore parents’ own thoughts and feelings about the perinatal experience. SSIs also allowed participants to share any additional information that they may not have felt comfortable discussing with other family members present in the CFI. During the field visit, we observed that homes were generally multi-generational, small, and in close proximity to neighbors. As the home setting affords little privacy, we conducted these SSIs in a private room at Hope Field Hospital when possible.

#### 3.2.3 Life story board (LSB) activity with children

To engage children in the research process, we invited children (age 7 + years old) from the families to participate in a play-based research activity that utilized the Life Story Board (LSB). The LSB is a developmentally appropriate method designed to explore children’s everyday lives, including their understanding of adversity and wellbeing, through their own narration (Chase et al., 2010; Stewart-Tufescu et al., 2019). The LSB approach was initially designed for use with war-affected children in Sri Lanka, and it has since been used in research with children in other humanitarian settings such as China, Cambodia, and northern Uganda (Chase, 2000; Chase et al., 2010). The LSB uses sets of cards, markers, and notation on a playboard to create a visual representation of the child’s life situation that is then narrated by the child during the storyboarding process. The recorded audio of the LSB interviews with the children revealed rich data on Rohingya children’s understanding of their own wellbeing, and specifically, in the context of the impending birth of a new sibling. The second author and a cultural liaison conducted the LSB with each family that had an eligible child willing to participate.

#### 3.2.4 Data collection process

Data gathering activities were conducted over two days. On Day 1, we traveled to the family’s home to complete the informed consent process and CFI. At the end of the interview, we presented the family with a gift to acknowledge our appreciation of their participation. The gift typically included some combination of fresh fruit, crackers, cookies, milk, candies/candy bars, soap, a printed Quranic verse wall hanging, and some small toys for the children. On Day 2, the SSIs and LSB were completed at Hope Field Hospital and scheduled on a day of the family’s choosing. This schedule was intended to ease the burden on mothers who are typically busy with household chores and meal preparation in the shelter, to accommodate fathers who were engaged in community and religious commitments, and children’s school attendance. Family members who traveled to the field hospital on Day 2 were offered a meal when they were at the clinic.

In keeping with best practices for ethical qualitative research in crisis-affected settings, the research team also implemented several strategies to address emotional distress that occasionally emerged during the interviews. As documented in previous work with displaced families, many participants were eager to share their stories and often described the process as meaningful or even cathartic despite moments of sadness or grief (Akesson et al., 2018). During all CFIs, SSIs, and LSB activities, participants were reminded that they could pause, take a break, skip any question, or end the interview entirely if the discussion became too distressing. The research team was led by two clinically trained social workers who were skilled in supporting families through sensitive conversations, monitoring signs of discomfort, and responding with trauma-informed practices. When emotional distress arose, interviews were slowed or temporarily stopped, and participants were gently offered space and choice regarding whether to continue. Consistent with findings from prior research in displacement contexts, many families chose to proceed, noting that being heard and having the opportunity to share their experiences was important to them. This flexible, participant-led approach ensured that emotional wellbeing was prioritized throughout all stages of data collection.

### 3.3 Analyzing research data

Consistent with recommended and commonly used practices for conducting cross-language research (Shklarov, 2007; Squires, 2009; Williamson et al., 2011) and with participants’ permission, all interviews, including the LSB interviews with children, were audio-recorded, capturing the English-speaking interviewers, the English-to-Rohingya translation, the Rohingya response, and the Rohingya-to-English translation. The specific Rohingya cultural liaison who assisted during the interview was also tasked with translating and transcribing the audio recordings for that particular family to produce an accurate set of English transcripts. All transcripts were checked and finalized by another Rohingya research assistant at the first author’s university to ensure data integrity and completeness.

LIPA involves detailed analysis of individual cases over time, followed by a search for similarities and differences between cases (Smith et al., 2009). To begin our data analysis, we held a series of discussion meetings with research team members, student research assistants, and cultural liaisons. These discussions were based on team members’ reading of a set of transcripts for a family and then sharing their impressions of the content, including participants’ articulated thoughts, feelings, and reflections on their experience of the perinatal period. Group notes taken during these discussions informed the thematic coding structure, which was used to create a codebook to subsequently apply to the qualitative interview data.

Codes were organized thematically within the broad categories of perinatal experiences, health (all types), family, community, education, environment, household economy, futures, and contextual factors. Within each broad category, subcodes were grouped temporally by the family’s experiences in Myanmar, their journey to Bangladesh, and their life displaced in Bangladesh. We then utilized Dedoose,^1^ a qualitative research data software program, to apply codes to interview transcript content. Two social work student research assistants (RAs; one master’s level and one undergraduate) coded the data under the supervision of the first and fourth authors. Interviews (CFIs, SSIs from mother and fathers, and LSBs with children) from each family were randomly assigned to the two RAs so that all families’ data were coded by more than one RA. Along with the fourth author, the two coders shared an analytic journal in which they documented and discussed discrepancies in the application of codes. Following the captured discussion, an agreed upon decision was noted. This journal along with the codebook guided the research team’s shared understanding and application of codes to the interview data. The coded content was then analyzed in aggregate by the authors to highlight the most salient issues and experiences. This paper focuses on the broad categories of perinatal experiences, family, and household economy related to subcodes specific to parent and child wellbeing, resource scarcity and allocation, and family dynamics during pregnancy.

### 3.4 Describing participating families

Study participants included 32 Rohingya families who had fled Myanmar during the 2017 genocide and were currently living in refugee camps in Cox’s Bazar, Bangladesh. See Table 1 for family demographic information including ages of family members, number of pregnancies, and number of living children.

**Table 1:** Description of participating families.

	*n*	Average	Percentage	Range
Age (in years)				
Mothers	32	27.1		17–56
Fathers	31	32.5		19–61
Children (female)	36	5.9		1.5–16
Children (male)	46	8.5		0.5–24
Number of families living with extended family members	14		43.6	
Number of children in the family				
None	5		15.6	
One	3		9.4	
Two	8		25.0	
Three or more	16		50.0	
Number of previous pregnancies	26	2.9		0–8

The average age of father participants (*n* = 31) was 32.5 years, ranging from 19 to 61 years old based on their verbal responses when asked their age. Participating mothers (*n* = 32) were slightly younger, with an average age of 27.1 years when verbally asked to report their age, with a range of 17 to 56 years old. The average age of female children (*n* = 36) was 5.9 years ranging in age from 1.5 to 16 years. Male children (*n* = 46) had an average age of 8.5 ranging from six months to 24 years old.

Sixteen of the 32 families (50.0%) we interviewed had three or more children. Fourteen families (43.6%) had extended family members living with them in the shelter. This included mothers- or fathers-in-law, uncles, aunts, and cousins. There were five families who had no previous children and were pregnant with their first child at the time of our visit. The average number of previous pregnancies (*n* = 26 mothers) was 2.9 ranging from no previous pregnancies to eight pregnancies. Not all pregnancies resulted in live births. Mothers were all pregnant when we visited, and even though our design was to interview pregnant women at approximately six months along, the length of gestation varied greatly in the verbal responses (from 1.5 months to nine months), as mothers and fathers often did not know the due date for the pregnancy. When available, we consulted corroborating documentation such as hospital visit records or birth attestation cards of parents to confirm the ages of participants and pregnancy gestation.

## 4 Results and discussion

The data resulted in three emergent themes, all aligning with our critical theoretical framework that understands the nurturing care (World Health Organization (WHO), 2018) of parents as existing within, being limited by, and sometimes also growing from the family’s experience of violence, repression, and displacement: (1) Parental wellbeing, which has direct implications for child wellbeing, (2) Resource scarcity and allocation, and (3) Shifting family dynamics. (Note: All participant names below are pseudonyms.)

### 4.1 Theme 1: parent–child wellbeing

Raising children in the Rohingya refugee camps in Bangladesh places extraordinary strain on caregivers, many of whom continue to endure the psychological trauma of fleeing genocide and the ever-present and increasing material impacts of displacement. Across interviews, parents described how the transition from Myanmar to camp life has led to profound emotional distress, exhaustion, and uncertainty about their children’s futures.

Mothers and fathers voiced deep anxiety about the implications of raising children in an environment marked by restriction and deprivation. 48-year-old father of six, Majid Naseem, reflected: “There is a big difference. Raising children in the camp is much more difficult… We do not want to have a baby here because we do not have any property here to leave for children” (Family 24 Dad SSI). Similarly, 26-year-old mother of two, Rafia Khatun, expressed fears about her children’s wellbeing in the absence of a support network: “I’m worried about my children’s future since I have no one on my side, which makes me feel like an orphan… I just wonder who will take care of my children if I become bedridden” (Family 8 Mom SSI).

The physical and emotional demands of caregiving were compounded by the material conditions of camp life. 25-year-old mother of three, Nafeesa Banu, explained: “If the baby is born in the hospital, we receive medical treatment and vaccinations… But if the baby is born at the shelter, we do not receive anything… I have to manage everything by myself” (Family 27 Mom SSI). Nafeesa Banu continued to emphasize the burden of raising children without basic services or family help, describing days filled with housework, worry, and caregiving tasks in isolation:

“We will have to raise the child while facing many difficulties, along with managing household chores… serve them food, help them bathe, wash clothes, take care of my husband and look after myself, all while caring for the new baby” (Family 27 Mom SSI).

Several mothers described the added complexities of simultaneously caring for extended family members (particularly mothers-in-law) while caring for their existing children. These responsibilities intensified perinatal stress, especially when compounded by fears of requiring medical interventions such as caesarean sections. Women worried that such medical procedures would prevent them from performing essential domestic and caregiving work, potentially leading to criticism or rejection from in-laws. In some cases, participants expressed deep anxiety that an inability to fulfill these domestic roles could prompt mothers-in-law to pressure their husbands to take an additional wife. These accounts underscore how health concerns in the perinatal period are deeply entangled with family power dynamics, gender expectations, and the precariousness of marital stability in displacement contexts.

For many parents, the pressure to provide material needs (e.g., food, healthcare, education) was a source of guilt and distress, especially when their health or economic status limited their ability to do so. 25-year-old father of three, Haroon Zaman, explained: “A father should also ensure that his children play and enjoy life… I cannot afford to buy toys for my children… My children are sad, because I am sad due to my illness” (Family 11 Dad SSI).

Despite these challenges, parents often linked their own wellbeing to the future aspirations they held for their children, especially around education. Consider the following quotes from three fathers:

22-year-old Abdul Mansur, who is expecting his first child: “I will try to educate them so that they do not have to do labour work like me… We left the country we had… Now we are living on the land of others” (Family 21 Dad SSI).

34-year-old father of three, Anwar Bashir: “I am thinking that if we have to continue living in the camp… we won’t be able to educate them… Thus, I am hoping that if we resettle to a third country, we will be able to educate them” (Family 14 CFI Dad).

26-year-old father of two, Abdul Karim: “If I can't provide them good food, then my children are also not happy… If I cannot provide them healthy food, then the child will not do well in education” (Family 1 Dad SSI).

At the same time, the systematic and structural limitations of life in the camp—such as unemployment restrictions and fear of arrest—left parents feeling powerless. 18-year-old Sakina Begum, pregnant with her first child, described: “I am always worried about how I will feed and provide for my children… If the police find someone leaving the camp to work, they are detained… So how will we provide for our children” (Family 21 Mom SSI)?

Parents described coping with adversity through faith and patience, though often with resignation. As father Majid Naseem explained: “If we do not have the things we want, we look at the ground and stay patient… We do not have any option to go anywhere… So, we have to live here” (Family 24 CFI). Together, these data underscore how deeply interconnected parental wellbeing is with children’s daily lives. Parents bear not only the trauma of the past and the uncertainty of the present, but also a heavy emotional and mental load tied to their children’s futures, especially related to educational opportunities. While many demonstrate resilience through expressed hopes and dreams for their children’s futures, the chronic stressors of camp life significantly erode parents’ sense of agency and empowerment, with real consequences for their caregiving capacities.

Mothers tended to describe wellbeing in terms of physical exhaustion, daily caregiving pressures, and the emotional labor of maintaining family stability. Fathers more often emphasized material and moral responsibilities related to provision. Older children (especially girls) articulated emotional consequences of parental distress, including the fear of losing parental affection or increased responsibility after the new baby’s arrival.

These findings directly reflect the interdependence of the five domains of nurturing care outlined in the World Health Organization (WHO) (2018) NCF: good health, adequate nutrition, responsive caregiving, opportunities for early learning, and safety and security. Rohingya parents’ ability to provide responsive caregiving was often compromised by structural barriers that also undermined nutrition, healthcare access, and safety, demonstrating how deficits in one domain reverberate across the whole caregiving environment.

### 4.2 Theme 2: resource scarcity and allocation

For these Rohingya families living in conditions of protracted displacement and extreme poverty, the impending arrival of a new child amplifies already existing pressures on limited resources. Parents described how food, space, money, and shared attention among their existing children are further strained, creating additional emotional turmoil, logistical burdens, and a pervasive sense of guilt about their caregiving capacity.

Many families anticipated that with each additional child, the demands on their time, energy, and resources would increase sharply. Father Haroon Zaman reflected on the impact of his growing household: “Now, if I bring half a kilo of fish, five of us eat it. In the future, we may need 750 grams or even one kilo of fish… Thinking about all of this, I feel worried” (Family 11 Dad SSI).

The sense of escalating scarcity weighed heavily on both mothers and fathers, as they contemplated how to maintain equal and consistent care for their children. Mother Rafia Khatun explained: “There will be four children. We cannot provide something bigger to one child and smaller to another… How can I fulfill the needs of all four children?” (Family 8 Mom SSI).

26-year-old mother of three, Farzana Banu explained, “When the baby arrives, we will be both sad and worried… We are unable to provide that kind of space for the baby… I am worried about whether I will be able to feed my baby with an alternative to breastfeeding” (Family 11 Mom SSI). Her husband, Haroon Zaman, added, “If the baby falls sick and the hospital cannot treat it, I will need money for private treatment. This is a constant worry for me” (Family 11 Dad SSI).

Some parents linked scarcity directly to the systemic and structural limitations of camp life, describing how employment restrictions and cramped shelter conditions compounded their feelings of parental inadequacy. This is captured in the following two quotes from mothers:

21-year-old mother of one, Sumaiya Khatun: “If we have money, we can go to the market to buy basic things for the children… When my husband goes out to look for a job, he is unable to go outside of the camp due to restrictions” (Family 31 Mom SSI).

19-year-old first-time mother, Rabia Banu: “Our shelter is very small with limited space, so how will we manage and adjust when we have more children?” (Family 23 Mom SSI).

In these contexts, parents often experienced a crisis in confidence, or an internal conflict between their deep sense of parental responsibility and the realities of resource scarcity. As father Abdul Mansur expressed: “I cannot raise my children the way our parents raised us… We are struggling to make a living” (Family 21 Dad SSI). And yet, many continued to articulate powerful aspirations for their children’s wellbeing, particularly around education and hope for resettlement. 30-year-old mother of four, Hameeda Khatun, said, “We are thinking how we will take care and provide basic needs… how we will feed and educate them because we lack money” (Family 22 CFI Mom). Her husband, 40-year-old Zubair Faruq added, “If I am unable to educate them due to financial problems despite working hard, I will beg to provide them with an education” (Family 22 CFI Dad).

Several parents described resorting to selling their monthly food rations or other essential items to cover the costs of their children’s education. These sacrifices were often made in the context of severe resource scarcity and restrictive camp policies that limit formal employment and restrict access to education beyond Grade 3. Such decisions underscore how parents’ aspirations for their children’s futures are shaped and constrained by broader structural and political forces. In these circumstances, the burden of financing education falls almost entirely on families themselves, reinforcing cycles of deprivation and deepening the trade-offs between meeting immediate nutritional needs and investing in long-term opportunities for their children. These accounts point to the ways in which systemic and policy-level barriers perpetuate the very conditions that parents struggle to overcome. The trade-offs parents described—such as sacrificing food security to finance education—underscore how interconnected the domains of the NCF (World Health Organization (WHO), 2018) are and also how much they are impacted by protracted displacement contexts. When the structural constraints of life after fleeing violence limit early learning opportunities, they also jeopardize nutrition, responsive caregiving, and overall safety, making it impossible for refugee families to fully realize the holistic nurturing care environment envisioned by the framework.

The political and structural constraints were not only recognized by parents but also by their children. For example, 12-year-old Amin Habib (Family 19 LSB) expressed that he did not think his parents should have more children because they could not afford to care for the ones they already have. His comment reflected both an awareness of the family’s economic limitations and a protective concern for his siblings’ wellbeing. This intergenerational perspective underscores how scarcity and structural barriers influence not only parental decision-making but also children’s own views on family size and future aspirations.

Mothers frequently focused on food distribution and the strain of meeting all their children’s needs, while fathers more often emphasized the frustrations of restricted employment and the inability to provide materially. Older daughters commented on the emotional implications of resource sharing, whereas younger children focused on concrete limitations such as ineffective schooling.

Notably, children themselves revealed an acute awareness of how resource limitations affect caregiving and availability of emotional support and love. 14-year-old Sajida Mahmud shared her mixed emotions about her parents having another child:

“When the new baby come[s], the loves of our mother will be shared among us. That’s the reason my mother will not love me once the new baby is born… I feel a bit happy because I can play with the baby… then I will be sad” (Family 7 LSB).

This quote, echoing a complex blend of pride, sacrifice, and emotional vulnerability, highlights how children internalize the tensions between scarcity and care. In doing so, it illustrates the emotional ripple effects of constrained parenting capacity, and how children too, become active participants in managing scarcity through compromise and hope.

### 4.3 Theme 3: shifting family dynamics

In the context of the Rohingya refugee camps, the profound scarcity of resources and physical infrastructure of the camps has led to a reshaping of traditional Rohingya family roles and responsibilities. Fathers, unable to fulfill their socially expected roles as breadwinners due to restrictions on movement and formal employment, expressed deep distress. Majid Naseem explained that although his wife had asked for her wedding gold (*mahr*):

“I don’t have money to buy it for her. I tried to convince her that when our children grow up, they might be able to buy it for her. Unfortunately, I had to sell her *mahr* because there is no source of income in the camp” (Family 24 Dad SSI).

This inability to provide has often shifted the economic burden onto mothers, who engage in creative, informal coping strategies. Mother Farzana Banu said, “After selling a liter of oil and Dal, we enrolled them in the school” (Family 11 Mom SSI), while 17-year-old first-time mother Zahida Khatun explained, “I sometimes sell one or two bottles of oil from the monthly ration to cover personal expenses” (Family 10 CFI Mom). These acts of economic ingenuity reflect both the moral burden of care and the gendered redistribution of survival labor within displaced households.

At the same time, the labor of caregiving is being reconfigured across generations, particularly among older children. In some families, older siblings actively contribute to household income and caregiving duties. 12-year-old Yasin Faruq described a scene from camp life: “I drew a picture of a shop because we had a shop in the camp where I used to sell snacks, juice, and other items” (Family 22 CFI). 10-year-old Nasir Kabir shared, “I go [to the market] alone. to buy vegetables, betel leaf, and betel nut” (Family 32 LSB). In one particularly telling case, 16-year-old first-time father, Musa Aziz, recounted being married for the main reason that his new wife could help care for his sick mother: “My mother was unable to cook. They made me marry to get help in preparing food” (Family 10 CFI Dad). These examples echo broader patterns of role reversal and childhood adultification that are well documented in contexts of displacement and political violence.

These caregiving responsibilities are highly gendered. Girls in particular are often tasked with both physical domestic responsibilities and also emotional labor. Hameeda Khatun noted that after the birth of her baby, she would rely on her husband and daughter to assist with water collection and housework (Family 22 CFI). Girls also expressed both pride and emotional ambivalence about these roles. 14-year-old Sajida Mahmud reflected, “I go to the market to buy fish, vegetables, and many other things” and acknowledged that while she looked forward to playing with her new sibling, she also feared that she would lose her mother’s affection once the baby was born (Family 7 LSB). This duality—of affection and anxiety—encapsulates the complex emotional terrain navigated by children, especially girls, who take on caregiving roles in times of crisis.

Despite these burdens, families also express adaptive strategies that illustrate resilience. 61-year-old father of three, Abdur Rahman spoke with hope that his children would “take care of me as I am taking care of them” (Family 2 Dad SSI), highlighting intergenerational reciprocity as a form of emotional and practical survival. 35-year-old father of two, Yasin Salim envisioned a future in which children would “take their own role in the family” (Family 6 CFI), revealing a shifting but still cohesive sense of familial obligation. These evolving roles are not simply imposed by crisis but are actively negotiated, demonstrating the fluid and adaptive nature of family life in displacement.

## 5 Summary of findings

Parenting in contexts of political violence and displacement requires navigating immense adversity while caring for children and anticipating the arrival of another child. This study found that political violence, displacement, and conditions within the refugee camps (such as food insecurity, restricted mobility, limited livelihood opportunities, and inadequate shelter) undermined parental efficacy and caregiving capacity. As previous literature confirms, chronic stress, trauma, and legal precarity can erode caregivers’ emotional wellbeing, leading to feelings of helplessness and guilt that interfere with responsive caregiving (Panter-Brick et al., 2014; Sousa et al., 2024). For Rohingya parents, the psychological toll is compounded by the structural violence of protracted encampment, where legal invisibility and dependency on humanitarian aid further constrain parental agency.

Despite these formidable challenges, the data also highlight the strength and resourcefulness of Rohingya families during the perinatal period. Many caregivers engaged in “amplified caregiving,” redoubling their emotional, spiritual, and practical efforts to protect their existing children and to also maintain familial stability for the arrival of a new baby. This aligns with existing research showing how parenting in adversity often entails intentional acts of love, storytelling, prayer, and resourcefulness that serve to preserve children’s sense of security and hope (Betancourt and Khan, 2008; Masten and Narayan, 2012). Children, too, contribute meaningfully to family functioning; older siblings assist with caregiving and economic tasks, and many express pride in these contributions to their family. However, these findings also illuminate missed opportunities for Rohingya children, namely limited access to formal education and vocational training due to restrictive camp policies. Together, these findings underscore the importance of understanding families not just as units of vulnerability, but also as interdependent systems of resourcefulness and care (Akesson, 2011).

Throughout the study, families demonstrated both compromised and adaptive caregiving modes in response to changing conditions that contradict traditional norms and practices. In this way, our study demonstrates the rich contributions to be realised by taking seriously the experiences and subjectivities of parents fleeing war and grappling with horrific displacement conditions. The creative redistribution of caregiving responsibilities—often falling on mothers and older daughters—reflects how family roles are reconfigured in crisis, echoing patterns seen in other war-affected contexts (El-Khani et al., 2016; Thabet et al., 2009).

These dynamics call for a socioecological approach that integrates individual, familial, and sociopolitical influences (Bronfenbrenner, 1979; World Health Organization (WHO), 2018). Recognizing the interconnectedness of caregiver wellbeing and child development, and the ways both are impacted by political and structural violence is essential for the establishment of understandings and interventions for refugee families. Family resilience cannot be sustained through individual fortitude alone; it requires dismantling the systemic and structural barriers—poverty, statelessness, and restrictive aid regimes and government policies—that shape everyday family life in contexts of forced displacement (Southwick, 2015).

## 6 Implications and conclusions

### 6.1 Practice and policy implications

The findings of this study have significant implications for humanitarian programming, service delivery, and policy formulation in Bangladesh’s Rohingya refugee camps as well as in other protracted displacement settings. First and foremost, there is an urgent need to design interventions that include psychosocial support for children and parents. While many child-focused services exist in the Rohingya camps, caregiver wellbeing is often under-addressed despite its critical influence on children’s developmental health and wellbeing. This oversight ignores the reality that parents are navigating profound psychological distress rooted in past trauma, daily insecurity, and the unrelenting demands of caregiving in extreme poverty. Integrated services that support parental mental health, including culturally appropriate counseling, peer support groups, and community-based psychosocial programming, are critical to bolstering caregivers’ resilience and indirectly improving child outcomes (Sousa et al., 2024).

Second, findings underscore the importance of recognizing and appropriately responding to older children’s caregiving roles within the family system. In the absence of external supports, many Rohingya children—particularly girls—take on significant domestic and emotional labor, contributing to family resilience but often at the expense of their own development and future opportunities. Interventions must therefore move beyond the narrow framing of children as passive recipients of care, instead acknowledging their active (though often overburdened) contributions to household functioning. Critically, programs and approaches must be targeted and age-appropriate, ensuring that children’s involvement in caregiving does not eclipse their rights to education, play, and psychosocial wellbeing (Betancourt and Khan, 2008; El-Khani et al., 2016).

These dynamics underscore the importance of recognizing extended family relationships as a critical yet complex component of the caregiving environment in humanitarian contexts. Models of family and child development and associated interventions in displacement settings should therefore consider not only the nuclear family but also the extended household structure when designing supports for maternal and child health. Culturally sensitive programming that addresses both the potential protective roles and the risks associated with in-law relationships could help mitigate conflict, strengthen social support systems, and ultimately enhance maternal wellbeing and newborn care in the critical perinatal period.

Finally, gender-sensitive programming is essential to address the disproportionate caregiving burdens placed on mothers and older daughters. Traditional gender norms—already strained by displacement—can become more rigid or more fluid in crisis contexts, requiring nuanced and responsive programming. Interventions should work with families to promote equitable caregiving responsibilities, engage fathers and male caregivers as active contributors to household wellbeing, and create safe spaces for girls to learn, play, and grow without being solely responsible for domestic labor. Community-based programs that engage both men and women in discussions of shared responsibility, dignity, and parenting under adversity have shown promise in other conflict-affected contexts (Tol et al., 2013).

The World Health Organization (WHO) (2018) NCF offers a valuable blueprint for holistic early childhood development. But in the Rohingya context, achieving its five interrelated components requires shifts in research and policy as well as service innovations. Without the kinds of enabling conditions our study reveals—such as access to education beyond Grade 3 and meaningful livelihood opportunities for refugee parents—the structural foundations of nurturing care remain incomplete. Aligning humanitarian and policy responses with the NCF would mean addressing these systemic barriers alongside psychosocial and health supports.

Overall, practice and policy must adopt a family systems lens, situated within a broader socioecological framework, that considers not only individual behaviors that impact caregiving and children’s developmental health, but also the broader political and structural conditions resulting from war and violence that constrain caregiving. Interventions must move beyond crisis-response to address the chronic conditions of structural violence that define refugee life, especially for most Rohingya families who have been residing in the camps since 2017. While many of these challenges can be mitigated through targeted humanitarian programming, lasting improvement in child and family wellbeing will require shifts in the broader environment. In particular, expanding opportunities for formal education beyond Grade 3 and creating safe, dignified pathways for livelihoods could empower families to meet both present and future needs. Though these shifts require careful coordination and long-term planning, they reflect shared goals of enhancing self-reliance, upholding human rights, and reinforcing human dignity.

### 6.2 Strengths and limitations

This study presents several methodological and conceptual strengths that enhance its contribution to the literature on caregiving in displacement. Most notably, the study uses multi-perspective data, incorporating the voices of mothers, fathers, and children, as well as broader family units through the CFI method. This approach centers the family as the unit of analysis and captures the intra-familial negotiation of care, burden, and hope. In particular, the inclusion of children’s voices through the LSB method enriches our understanding of how children themselves perceive and respond to shifting roles within the family. This child-inclusive approach aligns with growing calls in humanitarian and developmental research to recognize children not simply as passive recipients of care but as active participants in household resilience.

Despite these strengths, the study also has limitations. The current paper draws on Time 1 (approximately six months gestation) data only, representing a snapshot in the family’s longitudinal trajectory during the perinatal period. While the interpretive depth achieved through LIPA enhances contextual and experiential richness, the generalizability of findings is necessarily constrained. This is not a limitation of design but rather a reflection of its purpose: the goal of LIPA is not representativeness but deep, nuanced comprehension of fluid and evolving phenomena. In this case, we focused on how families make meaning of perinatal transitions in the context of chronic adversity, acknowledging that such meaning-making is both temporally situated and shaped by broader sociopolitical structures.

Although our methodological design and analytical process included attention to diversity across gender, age, and family role, the current manuscript focuses on shared thematic patterns observed across 32 participating families during Time 1. Because this paper draws on only the first wave of longitudinal data set, we intentionally limited subgroup comparisons to avoid overstating role-based differences at a single time point. Once longitudinal data collection and analysis are complete, we will examine how the perinatal transition may shape the different experiences of mothers, fathers, and children over time.

One of the most compelling promises of this research design is its potential to trace how caregiving changes within a static or deteriorating external environment. Even as humanitarian services fluctuate and camp conditions remain harsh, families themselves undergo normative transformations such as the birth of a child, the maturation of older siblings, or the shifting health of caregivers. This evolving landscape offers an opportunity to examine how care is restructured in response to both internal transitions and external constraints in family life. It also allows us to document how families strive to maintain “good enough” parenting amid scarcity, insecurity, and legal invisibility. In doing so, the study reinforces the value of longitudinal and ecological approaches for capturing the complexity of caregiving in contexts of displacement and political violence.

### 6.3 Future research directions

Although the current study is longitudinal in design—following Rohingya families over a two-year period during the perinatal stages—future research could extend this timeline to examine developmental trajectories across childhood and into adolescence or even adulthood. Longitudinal studies that span multiple developmental stages would allow researchers to better understand the enduring effects of parenting under chronic displacement on children’s emotional regulation, intellectual achievement, and identity formation. As Masten and Narayan (2012) emphasize, early adversity can have cascading effects across developmental domains, and understanding how caregiving practices mediate these pathways is essential for designing responsive interventions. Studies that continue to follow children into adulthood may also reveal how caregiving shapes intergenerational resilience or vulnerability under conditions of protracted displacement.

Our expanded discussion of father involvement in crisis settings reflects a growing recognition that perinatal care and family resilience cannot be solely understood through maternal narratives. Consistent with broader evidence showing that fathers’ emotional, practical, and mental health contributions shape maternal and infant outcomes (Firouzan et al., 2018; Lee et al., 2018; Small et al., 2025; Xie et al., 2023), our findings point to the need for research that continues to listen to fathers and other caregivers. While maternal caregiving has received considerable scholarly attention, fathers, older siblings, and extended kin also play key roles in navigating adversity and supporting children’s wellbeing. Prior research with refugee families has found that fathers often express care through protection, moral guidance, and spiritual instruction, particularly when traditional provider roles are disrupted (Akesson et al., 2020; Panter-Brick et al., 2014). Moreover, older siblings—especially girls—often engage in caregiving labor, taking on responsibilities that shape family resilience but may also curtail their own development. Future work should adopt a family systems approach that honors social-political realities, includes diverse caregiving configurations, and investigates how gender and age influence caregiving roles and burdens.

Finally, additional research is needed to examine how cultural and spiritual practices shape parental resilience and family wellbeing across displacement contexts. Refugee caregivers may draw upon religious beliefs, storytelling, music, and rituals as tools for emotional regulation, moral teaching, and creating a sense of continuity in disrupted environments (Betancourt and Khan, 2008). For example, recent work has demonstrated the cultural significance of traditional childbirth practices among Syrian refugees and how these rituals serve as both coping mechanisms and expressions of identity amid displacement (Alnaji et al., 2024). Future research could explore how such cultural assets can be mobilized within formal mental health and parenting interventions to enhance relevance and effectiveness in refugee settings.

## Data Availability

Data contain potentially identifiable and context-specific information and, to protect participant confidentiality, they will only be shared with others if the requesting researcher provides a detailed plan for maintaining participant confidentiality and obtains approval from an appropriate institutional ethics board. Requests to access the datasets should be directed to bakesson@wlu.ca.

## Footnotes

1
www.dedoose.com
